# The Use of eHealth for Pharmacotherapy Management With Patients With Respiratory Disease, Cardiovascular Disease, or Diabetes: Scoping Review

**DOI:** 10.2196/42474

**Published:** 2023-09-26

**Authors:** Robbert Bakema, Daria Smirnova, Despina Biri, Janwillem W H Kocks, Maarten J Postma, Lisa A de Jong

**Affiliations:** 1 Nederlandse Service Apotheek Beheer BV ’s-Hertogenbosch Netherlands; 2 Asc Academics Groningen Netherlands; 3 Erasmus School of Health Policy & Management Erasmus University Rotterdam Rotterdam Netherlands; 4 Victoria Hospital Kirkcaldy United Kingdom; 5 General Practitioners Research Institute Groningen Netherlands; 6 Groningen Research Institute Asthma and COPD University Medical Center Groningen University of Groningen Groningen Netherlands; 7 Observational and Pragmatic Research Institute Singapore Singapore; 8 Department of Pulmonology University Medical Center Groningen University of Groningen Groningen Netherlands; 9 Department of Health Sciences University Medical Center Groningen University of Groningen Groningen Netherlands; 10 Department of Economics, Econometrics and Finance University of Groningen Groningen Netherlands; 11 Center of Excellence in Higher Education for Pharmaceutical Care Innovation Padjadjaran University Bandung Indonesia

**Keywords:** telemedicine, polypharmacy, pharmacotherapy, diabetes mellitus, cardiovascular diseases, asthma, pulmonary disease, chronic obstructive, interventions e-health

## Abstract

**Background:**

eHealth is increasingly considered an important tool for supporting pharmacotherapy management.

**Objective:**

We aimed to assess the (1) use of eHealth in pharmacotherapy management with patients with asthma or chronic obstructive pulmonary disease (COPD), diabetes, or cardiovascular disease (CVD); (2) effectiveness of these interventions on pharmacotherapy management and clinical outcomes; and (3) key factors contributing to the success of eHealth interventions for pharmacotherapy management.

**Methods:**

We conducted a scoping review following the PRISMA-ScR (Preferred Reporting Items for Systematic Reviews and Meta-Analyses extension for scoping review) statement. Databases searched included Embase, MEDLINE (PubMed), and Cochrane Library. Screening was conducted by 2 independent researchers. Eligible articles were randomized controlled trials and cohort studies assessing the effect of an eHealth intervention for pharmacotherapy management compared with usual care on pharmacotherapy management or clinical outcomes in patients with asthma or COPD, CVD, or diabetes. The interventions were categorized by the type of device, pharmacotherapy management, mode of delivery, features, and domains described in the conceptual model for eHealth by Shaw at al (Health in our Hands, Interacting for Health, Data Enabling Health). The effectiveness on pharmacotherapy management outcomes and patient- and clinician-reported clinical outcomes was analyzed per type of intervention categorized by number of domains and features to identify trends.

**Results:**

Of 63 studies, 16 (25%), 31 (49%), 13 (21%), and 3 (5%) included patients with asthma or COPD, CVD, diabetes, or CVD and diabetes, respectively. Most (38/63, 60%) interventions targeted improving medication adherence, often combined for treatment plan optimization. Of the 16 asthma or COPD interventions, 6 aimed to improve inhaled medication use. The majority (48/63, 76%) of the studies provided an option for patient feedback. Most (20/63, 32%) eHealth interventions combined all 3 domains by Shaw et al, while 25% (16/63) combined Interacting for Health with Data Enabling Health. Two-thirds (42/63, 67%) of the studies showed a positive overall effect. Respectively, 48% (23/48), 57% (28/49), and 39% (12/31) reported a positive effect on pharmacotherapy management and clinician- and patient-reported clinical outcomes. Pharmacotherapy management and patient-reported clinical outcomes, but not clinician-reported clinical outcomes, were more often positive in interventions with ≥3 features. There was a trend toward more studies reporting a positive effect on all 3 outcomes with more domains by Shaw et al. Of the studies with interventions providing patient feedback, more showed a positive clinical outcome, compared with studies with interventions without feedback. This effect was not seen for pharmacotherapy management outcomes.

**Conclusions:**

There is a wide variety of eHealth interventions combining various domains and features to target pharmacotherapy management in asthma or COPD, CVD, and diabetes. Results suggest feedback is key for a positive effect on clinician-reported clinical outcomes. eHealth interventions become more impactful when combining domains.

## Introduction

eHealth is increasingly considered an important tool for supporting pharmacotherapy management. This is particularly emphasized at times when health care systems are under strain, such as during the SARS-CoV-2 pandemic, and more generally due to the increasing demands of aging populations, in which polypharmacy can be complex to manage [1,2]. eHealth is an umbrella term that encompasses different types of electronic technologies, both online and offline, as applied to the domain of health care, broadly defined. A commonly used definition of eHealth is: “The use of new information and communication technologies – in particular internet technology – to support or improve health and healthcare” [3].

Pharmacies play an important role in the safe and effective use of drugs in ambulatory settings. eHealth applications can help the pharmacist to improve the efficiency of health care by providing the patient with easily accessible information. eHealth can contribute to greater health care accessibility and quality while also empowering patients to take control of their disease and medication management by providing relevant and timely information [4,5]. eHealth, as applied to pharmacotherapy management, includes technologies for medication and prescription reminders, telemedicine, medication dispensing, providing information about medication, side effects, dosage, administration, and supporting patients and carers to manage their optimal administration [1,4,5].

Although there is growing interest in eHealth to support patients and health care providers in pharmacotherapy management, the question remains what type of interventions are most effective. eHealth is a broad concept, and many different types of interventions have been and continue to be developed. However, development and implementation are time-consuming and costly. With increasing numbers of eHealth interventions, it is relevant to explore the possibilities and limitations of eHealth interventions for pharmacotherapy management published to this point and look for lessons learned.

We performed a scoping review that aimed to provide foundational evidence for the current state of the landscape of eHealth use in pharmacotherapy management with patients with asthma or chronic obstructive pulmonary disease (COPD), cardiovascular disease (CVD), or diabetes. To this end, the following 2 questions were of importance: (1) How is eHealth used for pharmacotherapy management with patients with asthma or COPD, CVD, and diabetes? and (2) How effective are eHealth interventions for pharmacotherapy management and clinical outcomes? Subsequently, the type of intervention was linked to the effectiveness to answer the third question, which was (3) What key aspects make eHealth interventions for pharmacotherapy management successful? Our review focused on 3 common diseases that frequently require careful medication management and are major contributors to polypharmacy [6]. In particular, we considered respiratory disease (asthma and COPD), CVD (specifically hypertension and coronary artery disease), and diabetes mellitus, all involving large patient numbers, requiring careful management by a multidisciplinary team, and involving pharmacotherapy management as a cornerstone of care.

## Methods

### Study Design

We conducted a scoping review to identify relevant peer-reviewed publications on the use and effectiveness of eHealth interventions on pharmacotherapy management in the 3 selected patient populations of asthma or COPD, CVD, and diabetes. This scoping review was conducted and reported according to the PRISMA-ScR (Preferred Reporting Items for Systematic Reviews and Meta-Analyses extension for scoping reviews) statement [7]. This statement follows the same principles as the PRISMA statement for systematic reviews [8] but excludes some parts that are not relevant for scoping reviews. A search protocol was developed and revised using input from an advisory board of eHealth experts and clinicians. The final version of the protocol can be found in Multimedia Appendix 1.

### Domains of eHealth

The commonly used definition of eHealth is broad and lacks the specificity to describe the nature of the eHealth intervention. Therefore, we used the conceptual model by Shaw et al [9] to make the definition more usable for the scoping review. This conceptual model is based on semistructured interviews with key informants and contains the following 3 thematically analyzed emergent domains:

1. Health in our Hands (using eHealth technologies to monitor, track, and inform health care)
2. Interacting for Health (using digital technologies to enable health communication among practitioners and between health professionals and clients or patients)
3. Data Enabling Health (collecting, managing, and using health data)

**Figure 1:**
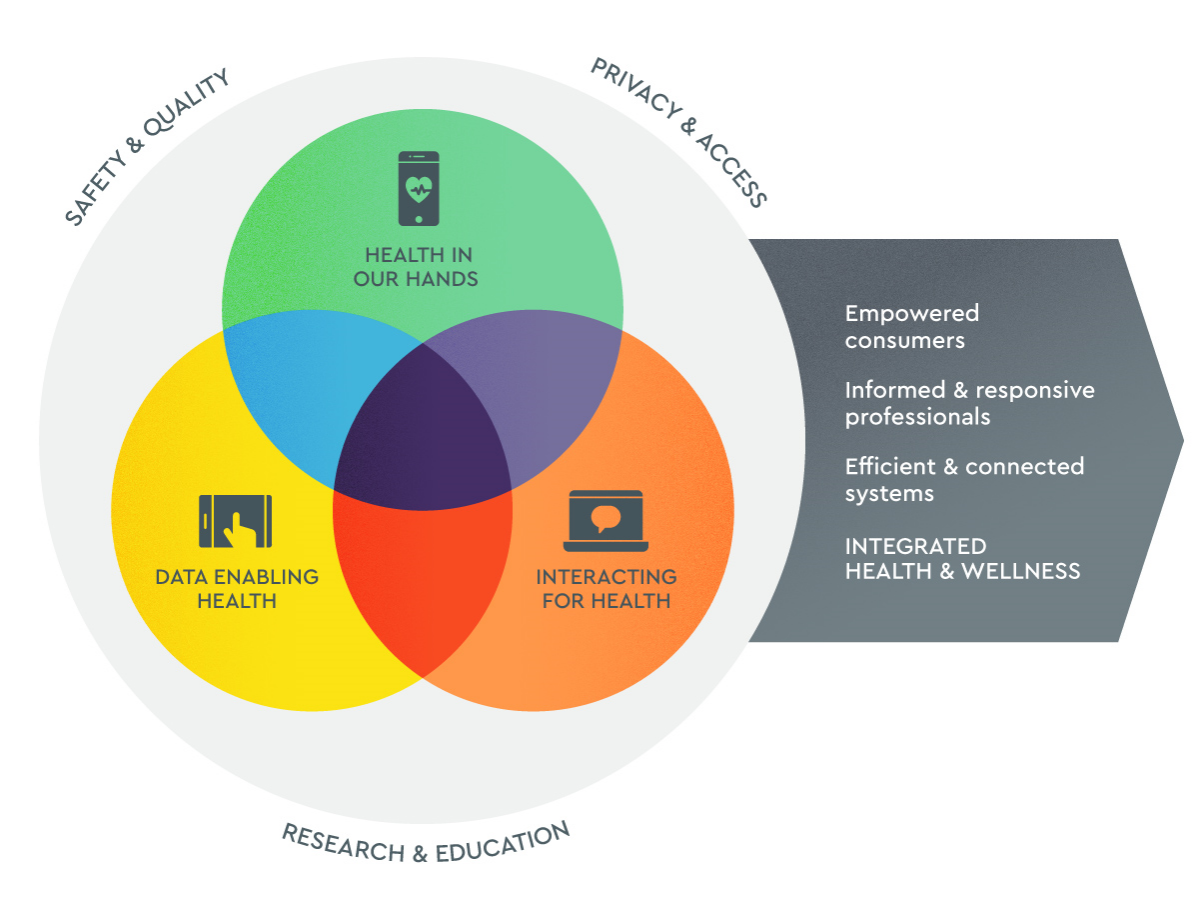
The conceptual model for eHealth developed by Shaw et al [9]. Figure reproduced with permission from Shaw et al [9].

These are described in Figure 1.

The eligibility criteria for the scoping review were based on a combination of the definition from the eHealth monitor 2019 [3] and the domains defined by Shaw et al. [9], with the intervention fitting at least in 1 of the 3 domains. The domains were also used to analyze the interventions.

### Eligibility Criteria

Search eligibility criteria were defined using the PICOS (Population, Intervention, Comparator, Outcomes, Study Design) framework. The framework is presented in Table 1. The population was limited to the 3 main indications with chronic medication use: asthma or COPD, CVD, and diabetes. The intervention had to support pharmacotherapy management directly or indirectly (eg, medication adherence or self-management of treatment) and should cover at least one of the domains defined by Shaw et al [9]. The comparator should be usual care. Included studies should at least include pharmacotherapy management or clinical outcome assessment. This could be either on patient- or clinician-reported clinical outcomes. Only randomized controlled trials (RCTs) and observational studies in Europe, North America, Australia, and New Zealand were considered for inclusion to limit the search to countries with similar economies. The search in the electronic medical databases was limited to the English language. The searches were performed without a date limitation.

**Table 1:** PICOS (Population, Intervention, Comparator, Outcomes, Study Design) criteria and language, time, and geographical limits for the inclusion and exclusion criteria for studies.

Category	Inclusion criteria	Exclusion criteria
Population (P)	Patients with asthma or COPD^a^ Patients with CVD^b^ Patients with diabetes mellitus	—^c^
Intervention (I)	eHealth interventions covering at least one of the 3 domains by Shaw et al [9] and directly or indirectly supporting pharmacotherapy management including medication adherence	—
Comparators (C)	Usual care	No comparator Versus baseline^d^
Outcomes (O)	Pharmacotherapy management or clinical outcomes	—
Study design (S)	RCTs^e^—both parallel-group and crossover (double-blind, single-blind, open-label) Retrospective and prospective cohort studies	In vitro studies Preclinical studies Comments, letters, and editorials Case reports and case series Guidelines Single-arm trials Reviews Meta-analyses
Language	English	Non-English
Time limit	No restriction	—
Country	Western countries: Europe United Kingdom United States Canada Australia New Zealand	—

^a^COPD: chronic obstructive pulmonary disease.

^b^CVD: cardiovascular disease.

^c^No exclusion criteria.

^d^Unless baseline represents usual care.

^e^RCTs: randomized controlled trials.

### Information Sources

The following electronic databases were searched: Embase, MEDLINE (PubMed), the Cochrane Library (including the Cochrane Database of Systematic Reviews and Cochrane Central Register of Controlled Trials). Reference lists of any identified trials, reviews, or meta-analyses of eHealth interventions were searched for further studies of interest.

### Search and Selection of Articles

Articles were initially searched up to March 23, 2020. The search was updated with articles from March 23, 2020, to October 1, 2021. The final Embase search strategy is presented in Multimedia Appendix 2. All retrieved studies were assessed against the eligibility criteria for the search. After manual deduplication with Zotero software (version 5.0), titles and abstracts were exported from Zotero to Rayyan format for screening purposes [10]. The study selection process was performed in 2 phases. First, to screen titles and abstracts, the titles and abstracts of studies identified from the electronic databases were double screened by 2 independent researchers (RB and LJ) to determine eligibility according to the inclusion and exclusion criteria. Second, to screen the full texts, the full texts of studies selected in the title and abstract screening were obtained and double screened by the same 2 independent researchers to determine eligibility. If there was disagreement on study selection in any of the screening phases, consensus was reached through a discussion with other authors if needed. The results of the article selection process were documented in a PRISMA flow diagram.

### Data Extraction and Synthesis

Data were extracted from full-text publications. The data charting table was agreed upon before populating the table. Data extraction was carried out by 1 researcher (DS). However, any uncertainty about the data was resolved in discussion with the 2 researchers who performed article selection (RB and LJ). Quality control procedures included verification of all extracted data with sources.

We developed a standardized Excel (Microsoft Corp) document for data extraction to tabulate specific information from the included studies. These included study and patient characteristics, type of intervention, and effectiveness outcomes. Study and patient characteristics (number of patients, study design, study duration, age, disease, study duration) were grouped by population (asthma or COPD, CVD, diabetes). To analyze what type of eHealth interventions are currently being used in the 3 disease areas of interest, the interventions were categorized by the type of device used (eg, robot, mobile application), pharmacotherapy management (eg, improve adherence, optimize treatment plan), mode of delivery (eg, education, telemonitoring, reminders, motivation), and (number of) domains as defined by Shaw et al [9]. The effectiveness on pharmacotherapy management outcomes and patient- and clinician-reported clinical outcomes was analyzed per type of intervention to identify trends. To indicate the impact of the eHealth intervention, interventions were scored as positive, neutral, or negative, in line with a previously published review on the effectiveness of eHealth [5]: positive: 1 positive outcome and no negative; neutral: no positive and no negative impact; negative: 1 negative outcome and no positive outcomes. An outcome was considered positive if the intervention showed a significant improvement compared with usual care. Similarly, an outcome was considered negative if the intervention was significantly worse than usual care. All other outcomes were considered neutral. Findings are presented descriptively and in tables and figures.

## Results

### Search and Screening Results

The initial searches were conducted on March 23, 2020, and were not limited by date. These searches yielded a total of 3352 records that were identified as unique by Zotero (Embase=1170, PubMed=1289, and Cochrane Library=893). In the updated search, from March 23, 2020, to October 1, 2021, an additional 1631 records were identified (Embase=749, PubMed=548, and Cochrane Library=334). After manual deduplication in Zotero, a total of 2483 (initial search) and 1297 (updated search) records were eligible for title and abstract screening. Titles and abstracts were exported from Zotero to Rayyan format for screening purposes.

**Figure 2:**
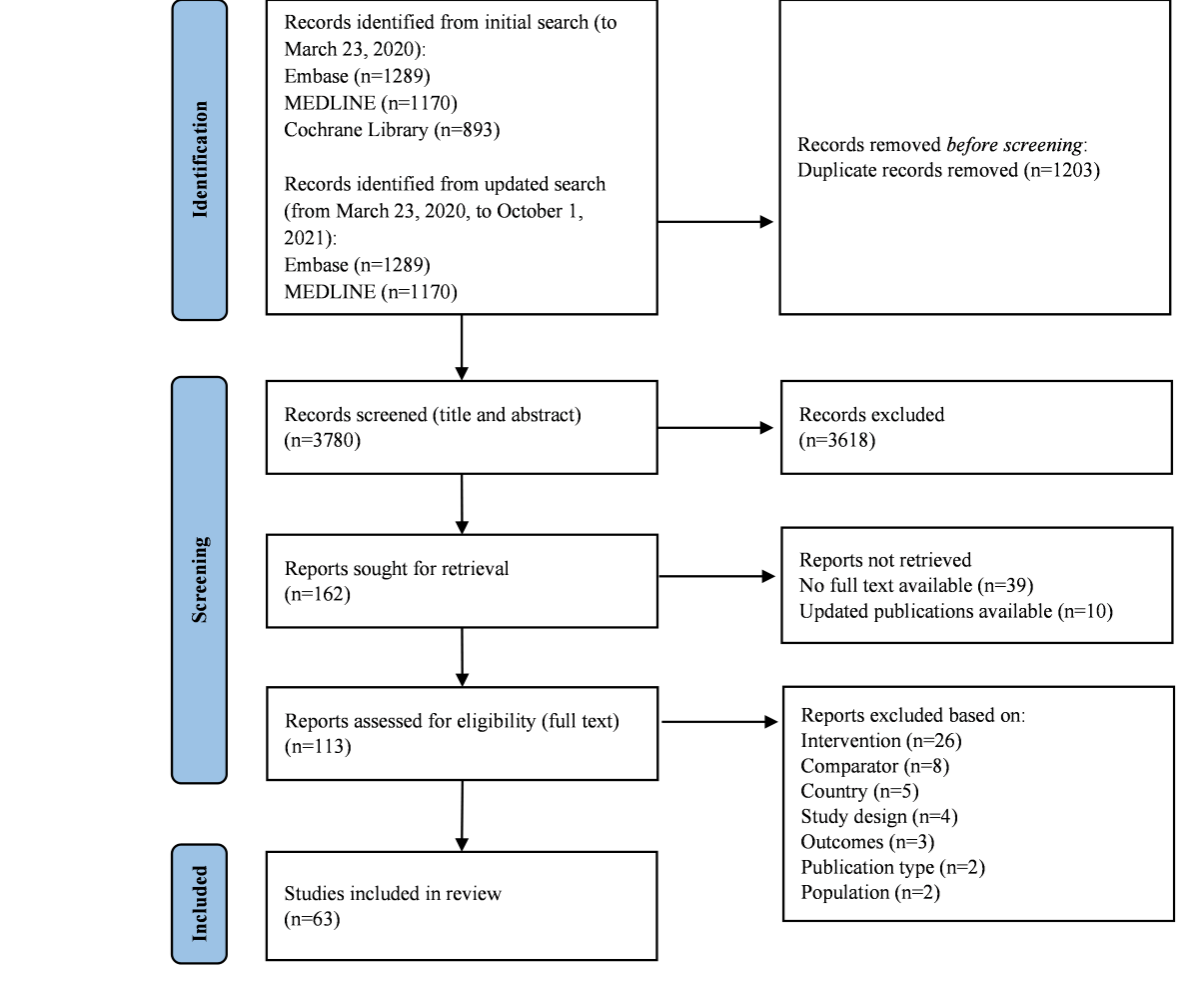
PRISMA (Preferred Reporting Items for Systematic Reviews and Meta-Analyses) flow diagram.

After screening of titles and abstracts, 162 publications progressed to full-text screening. Of the 162 publications, 39 were excluded because there was no full-text available (conference abstracts), and 10 articles were excluded because updated articles or more relevant analyses for this scoping review were available. During full-text screening, 63 articles met the predefined inclusion criteria and thus were selected for data extraction. The main reasons for exclusion at full-text screening were that the intervention, comparator, or country did not meet the inclusion criteria. The volume of articles included and excluded at each stage of screening is shown in the PRISMA flow diagram presented in Figure 2. Multimedia Appendix 3 presents the 63 articles ultimately included in the review and used to extract data.

### Study and Patient Characteristics

Most studies (42/63, 67%) were conducted in the United States. The other 33% (21/63) of the studies were conducted in a variety of European countries (including the United Kingdom, Netherlands, Sweden, Spain), Australia, and New Zealand. Most (59/63, 94%) studies were RCTs, of which 18 (29%) had a pilot, feasibility, quasi-experimental, or pragmatic study design. Of the remaining studies, 3 studies were nonrandomized trials, and 1 was an observational study. The study duration varied from 2 weeks to 54 months, although most (52/63, 83%) studies had a duration of 3 months to 12 months. There were no specific trends observed in the study characteristics for the 3 populations.

Among the 63 studies included in the analysis, patients with CVD constituted the largest group, accounting for 49% (31/63) of the total. Hypertension was the most (18/31, 58%) prevalent condition within this subgroup, followed by CHD and heart failure (9/31, 29%) [11-41]. The second largest group consisted of patients with asthma or COPD, representing 25% (16/63) of the studies. Within the respiratory disease category, the majority (14/16, 88%) were aimed specifically at patients with asthma, while only 2 studies specifically targeted patients with COPD [42-56]. Furthermore, only the study by O’Dwyer et al [57] included both asthma and COPD patients. Patients with diabetes comprised 21% (13/63) of the included patients, with the majority (9/13, 69%) of studies targeting patients with type 2 diabetes [58-66]. The remaining diabetes studies focused on type 1 diabetes or uncontrolled diabetes [67-70]. Additionally, 3 studies included a population consisting of patients with both hypertension and type 2 diabetes [71-73].

An overview of the distribution of eHealth trials among the 3 disease groups is presented in Table 2. The sample sizes across these studies varied considerably, ranging from 10 to nearly 15,000 patients. Notably, the mean age of patients with asthma was lower compared with the other populations, predominantly due to the inclusion of children and adolescents in most of the studies, in contrast to the other studies that exclusively included adult patients.

**Table 2:** Population—distribution of eHealth trials among the 3 disease groups of interest.

Disease groups			Patient distribution, n (%)
**Asthma or COPD^a^(n=16)**			
	Asthma	13 (21)	
	COPD	2 (3)	
	Both	1 (2)	
**CVD^b^(n=31)**			
	Hypertension	18 (28)	
	CHD^c^	7 (11)	
	Heart failure	2 (3)	
	Atrial fibrillation	2 (3)	
	Mix	1 (2)	
	Patients on OAC^d^	1 (2)	
**CVD and diabetes (n=3)**			
	Hypertension and diabetes	3 (5)	
**Diabetes (n=13)**			
	Type 2 diabetes	9 (14)	
	Type 1 diabetes	2 (3)	
	Uncontrolled diabetes	2 (3)	

^a^COPD: chronic obstructive pulmonary disease.

^b^CVD: cardiovascular disease.

^c^CHD: coronary heart disease.

^d^OAC: oral anticoagulant.

### How is eHealth Used for Pharmacotherapy Management?

We identified 3 types of pharmacotherapy management goals: medication adherence, medication use, and treatment plan optimization (Table 3). In the total population, most (38/63, 60%) interventions were targeted at improving medication adherence, which was often, but not always, combined to optimize the treatment plan. In addition, 6 interventions were designed to improve medication use, of which all concerned interventions for patients with asthma or COPD to improve inhaled medication use (frequency and inhaler technique). For patients with diabetes with or without CVD, most interventions were used to optimize the treatment plan.

Pharmacotherapy management goals could be achieved through different features of the interventions. Most (45/63, 71%) interventions had a combination of different features. Most (38/63, 60%) interventions aimed to improve pharmacotherapy management through monitoring of disease control (eg, blood pressure for patients with hypertension or asthma control testing), followed by features of education about medication or disease. Monitoring of medication adherence was also frequently (18/63, 29%) used, especially in asthma/COPD and CVD + diabetes populations, while none of the interventions for the diabetes population used the option of medication adherence monitoring to improve pharmacotherapy management.

Most (48/63, 76%) interventions provide an option for feedback to the patient. Feedback was delivered either through eHealth as part of the intervention or through non-eHealth communication systems (ie, not integrated in the eHealth intervention itself). Some interventions offered the option to provide feedback through multiple forms of communication. Overall, most (30/63, 63%) studies provided feedback through non-eHealth communication systems, specifically telephone or email, on information collected through the eHealth intervention. Of the 48 studies that provided feedback to the patient, 23 (48%) provided the option to give feedback through the eHealth intervention itself, of which most (19/23, 83%) were delivered through an application or platform. Trends were similar across the disease populations, except for the CVD + diabetes population, for which feedback in all 3 studies was provided through the eHealth application or platform itself.

**Figure 3:**
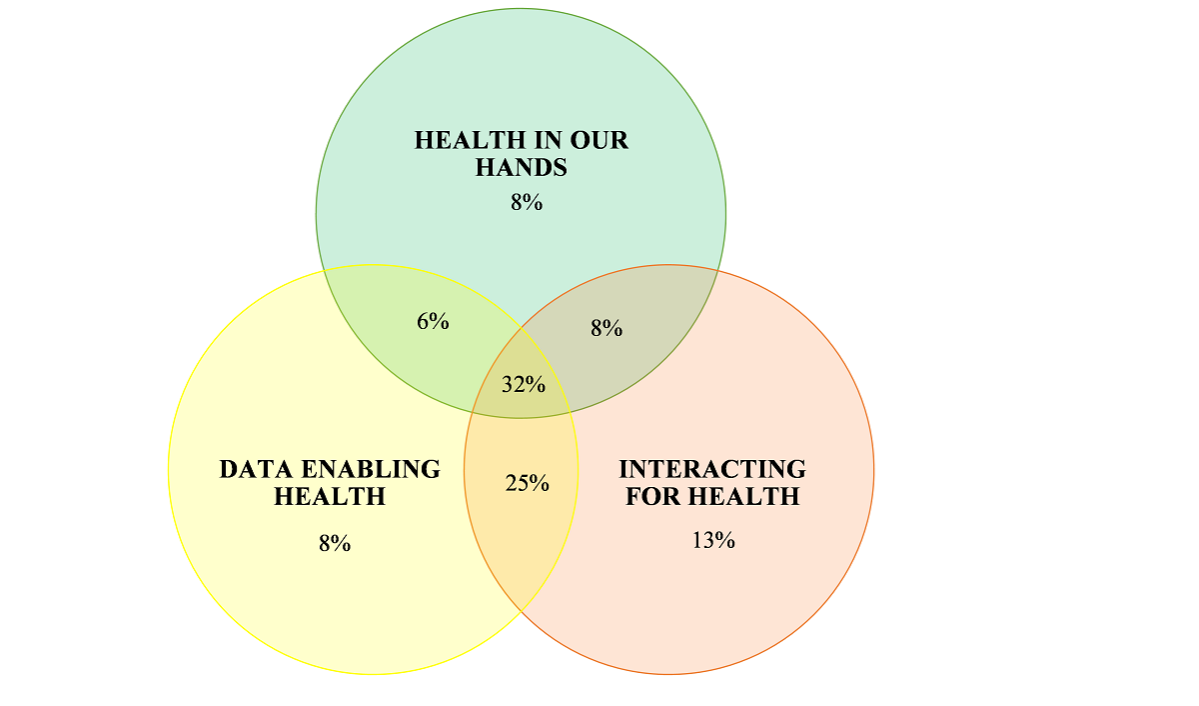
Intervention—classified by the domains by Shaw et al [9].

The types of intervention were also classified by the domains from the conceptual model by Shaw et al [9] (Table 3). In the total population, most eHealth interventions (20/63, 32%) combined all 3 domains, followed by 25% (16/63) combining Interacting for Health with Data Enabling Health. Interventions covering both the Health in our Hands and Data Enabling Health domains represented the smallest sample (4/63, 6%). The results were comparable across the populations. Figure 3 is a visual representation of the distribution around the domains by Shaw et al [9].

Of the total number of studies, 42 out of 63 (67%) showed a positive overall effect of the eHealth intervention (Table 3). Only one study showed a negative effect. In the CVD and diabetes populations over 70% of the studies showed a positive effect. In the population with comorbidity of CVD and diabetes all three included studies showed a positive effect. However, for the asthma or COPD population, only 8 out of 16 (50%) of the studies showed a positive effect, while the remaining 50% showed a neutral effect.

**Table 3:** Intervention classified by type of pharmacotherapy management, feedback, domains by Shaw et al [9], and overall effect.

Classification			Total number of studies (n=63), n (%)	Asthma or COPD^a^ (n=16), n (%)	CVD^b^ (n=31), n (%)	CVD + diabetes (n=3), n (%)	Diabetes (n=13), n (%)
**Pharmacotherapy management goals**							
	Medication adherence		38 (60)	8 (50)	23 (74)	2 (67)	5 (38)
	Medication use		6 (10)	6 (38)	0	0	0
	Optimize treatment plan		31 (49)	7 (44)	12 (39)	3 (100)	9 (69)
**Features**							
	Monitoring medication adherence		18 (29)	7 (44)	9 (29)	2 (67)	0
	Monitoring disease control		38 (60)	8 (50)	18 (58)	2 (67)	10 (77)
	Monitoring medication use		5 (8)	5 (31)	0	0	0
	Education about the disease		25 (40)	4 (25)	14 (45)	2 (67)	5 (38)
	Education about the medication		26 (41)	5 (31)	17 (55)	0	4 (31)
	Motivation		5 (8)	2 (13)	2 (6)	0	1 (8)
	Reminders		24 (38)	7 (44)	13 (42)	2 (67)	2 (1)
	Access to personal information		6 (10)	3 (19)	2 (6)	0	1 (8)
**Feedback to patient**							
	Yes		48 (76)	13 (81)	23 (74)	3 (100)	9 (69)
	No		15 (24)	3 (19)	8 (26)	0	4 (31)
**Mode of feedback delivery**							
	Feedback through eHealth		23 (48)	6 (46)	10 (43)	3 (100)	4 (44)
	**Mechanism of feedback delivery through eHealth**						
		Application or platform	19 (40)	5 (38)	8 (35)	3 (100)	3 (33)
		IVR^c^, automated telephone, or video call	4 (8)	1 (8)	2 (9)	0	1 (11)
	Feedback through non-eHealth		30 (63)	7 (54)	14 (61)	2 (67)	7 (78)
	**Mechanism of feedback delivery through non-eHealth**						
		Telephone/email	20 (42)	6 (46)	7 (30)	1 (33)	6 (67)
		In person	5 (10)	1 (8)	3 (13)	1 (33)	0
		Text message	3 (6)	0	3 (13)	0	0
		Printout	2 (4)	0	1 (4)	0	1 (11)
**Domains by Shaw et al** [9]							
	Number of studies covering 1 domain		18 (9)	3 (19)	13 (42)	0	2 (15)
	**Domain covered in studies covering 1 domain**						
		Interacting for Health	8 (13)	2 (13)	5 (16)	0	1 (8)
		Health in our Hands	5 (8)	0	5 (16)	0	0
		Data Enabling Health	5 (8)	1 (6)	3 (10)	0	1 (8)
	Number of studies covering 2 domains		25 (40)	8 (50)	9 (29)	1 (33)	7 (54)
	**Domains covered in studies covering 2 domains**						
		Interacting for Health + Health in our Hands	5 (8)	1 (6)	2 (6)	0	2 (15)
		Interacting for Health + Data Enabling Health	16 (25)	5 (31)	6 (19)	0	5 (38)
		Health in our Hands + Data Enabling Health	4 (6)	2 (13)	1 (3)	1 (33)	0
	Number of studies covering all 3 domains		20 (32)	5 (31)	9 (29)	2 (67)	4 (31)
**Overall effect**							
	Positive		42 (67)	8 (50)	22 (71)	3 (100)	9 (69)
	Neutral		20 (32)	8 (50)	8 (26)	0	4 (31)
	Negative		1 (2)	0	1 (3)	0	0

^a^COPD: chronic obstructive pulmonary disease.

^b^CVD: cardiovascular disease.

^c^IVR: interactive voice response.

### How Effective are eHealth Interventions on Pharmacotherapy Management and Clinical Outcomes?

Table 4 shows the effect of the eHealth interventions on pharmacotherapy management and clinical outcomes for the total number of studies. Pharmacotherapy management outcomes included medication adherence, change in medication prescriptions or use, inhaler technique, and knowledge about medication. These outcomes could either be patient- or clinician-reported. Patient-reported clinical outcomes included patient questionnaires on disease control, self-efficacy, disease and medication knowledge, and quality of life (including anxiety, depression, and physical activity questionnaires). Clinician-reported clinical outcomes mainly focused on disease control expressed as the number of hospitalizations (all diseases), exacerbations (asthma/COPD), blood pressure and lipid profile (CVD), and blood glucose levels (diabetes).

Of all included studies, 78% (49/63) reported clinician-reported clinical outcomes, and 76% (48/63) reported pharmacotherapy management outcomes. Patient-reported clinical outcomes were reported in 49% (31/63) of the included studies. Of the studies reporting pharmacotherapy management outcomes, 48% (23/48) reported a positive effect, 50% (24/48) had a neutral effect, and 2% (1/48) had a negative effect. Of studies reporting the clinician-reported clinical outcomes, 57% (28/49) reported a positive effect, 43% (21/49) reported a neutral effect, and none had a negative effect. Patient-reported clinical outcomes showed a positive effect in 39% (12/31) of the studies, while 58% (18/31) and 3% (1/31) reported a neutral or negative effect, respectively.

Two studies that reported a neutral effect for their overall study population, and therefore marked as neutral in our analysis, did show a significant positive effect on subpopulations. One study only showed a significant increase in medication adherence in the subpopulation with low baseline medication adherence [52], and 1 study showed only a significant improvement in asthma control in the subgroup of patients with 2 or more exacerbations before inclusion in the interventional group [55].

**Table 4:** Effect on pharmacotherapy management and clinician- and patient-reported clinical outcomes.

Studies and effect		Overall effect, n (%)	Pharmacotherapy management outcomes, n (%)	Clinical outcomes, n (%)	
				Clinician-reported	Patient-reported
Number of studies that reported the outcome		63 (100)	48 (76)	49 (78)	31 (49)
**Effect**					
	Positive	42 (67)	23 (48)	28 (57)	12 (39)
	Neutral	20 (32)	24 (50)	21 (43)	18 (58)
	Negative	1 (2)	1 (2)	0	1 (3)

### What Key Aspects Make eHealth Interventions for Pharmacotherapy Management Successful?

Table 5 shows the effect (positive, neutral, negative) of the eHealth interventions for pharmacotherapy management relative to the interventions’ number of features, number of domains by Shaw et al [9], and whether the intervention provided feedback to the patient for the total population. Of the interventions with 1 or 2 features for pharmacotherapy management, most studies (1 feature: 7/11, 64%; 2 features: 10/18, 56%) showed a neutral effect for pharmacotherapy management, while for interventions with ≥3 features combined, most (11/19, 58%) studies showed a positive effect. Clinician-reported clinical outcomes showed an opposite effect: Interventions with 2 features had a higher percentage of studies reporting a positive effect than interventions with 1 or ≥3 features. Patient-reported clinical outcomes were positive in 30% (3/9) and 40% (4/10) of the studies with an intervention with 1 or 2 features respectively, while 45% (5/11) of studies with an intervention with ≥3 features showed a positive effect.

The interventions covering 3 domains showed a positive effect on pharmacotherapy management in 64% (9/14) of the studies, while this percentage was lower for interventions covering 1 (7/16, 44%) or 2 (7/18, 39%) domains. There was also a trend toward a higher number of studies reporting a positive effect on clinical outcomes with an increasing number of domains by Shaw et al [9], from 42% (5/12) positive studies with 1 domain to 67% (10/15) with 3 domains for clinician-reported clinical outcomes and from 33% (2/6) to 36% (4/11) for patient-reported clinical outcomes. However, for these outcomes, there was not much difference between interventions combining 2 or 3 domains.

Of the interventions providing feedback to the patient, 46% (16/35) had a positive effect on pharmacotherapy management outcomes, while 54% (7/13) of the studies with an intervention without feedback had a positive result. This indicates that there is no clear effect of feedback on pharmacotherapy management outcomes. However, feedback does have an impact on clinical outcomes. Of the studies with an intervention with feedback to the patient, 66% (27/31) and 40% (10/25) reported a positive effect on clinician- and patient-reported clinical outcomes, respectively, while the proportions were 13% (1/8) and 33% (2/6), respectively, for interventions without feedback.

**Table 5:** Effectiveness relative to interventions’ number of features, number of domains by Shaw et al [9], and feedback to the patient.

Effectiveness				Number of features, n (%)							Number of domains by Shaw et al [9], n (%)							Feedback to patient, n (%)		
				1		2		≥3		1			2		3		Yes			No
**Overall effect**																				
	Positive			13 (68)		14 (67)		15 (65)		10 (56)			18 (72)		14 (70)		33 (69)			9 (60)
	Neutral			6 (32)		7 (33)		7 (30)		7 (39)			7 (28)		6 (30)		15 (31)			5 (33)
	Negative			0		0		1 (4)		1 (6)			0		0		0			1 (7)
**Pharmacotherapy management outcomes**																				
	Positive			4 (36)		8 (44)		11 (58)		7 (44)			7 (39)		9 (64)		16 (46)			7 (54)
	Neutral			7 (64)		10 (56)		7 (37)		8 (50)			11 (61)		5 (36)		19 (54)			5 (38)
	Negative			0		0		1 (5)		1 (6)			0		0		0			1 (8)
**Clinical outcomes**																				
	**Clinician-reported**																			
		Positive	9 (50)		10 (71)		9 (53)		5 (42)			13 (59)		10 (67)		27 (66)			1 (13)	
		Neutral	9 (50)		4 (29)		8 (47)		7 (58)			9 (41)		5 (33)		14 (34)			7 (88)	
		Negative	N/A^a^																	
	**Patient-reported**																			
		Positive	3 (30)		4 (40)		5 (45)		2 (33)			6 (43)		4 (36)		10 (40)			2 (33)	
		Neutral	7 (70)		5 (50)		6 (55)		4 (67)			7 (50)		7 (64)		14 (56)			4 (67)	
		Negative	0		1 (10)		0		0			1 (7)		0		1 (4)			0	

^a^N/A: not applicable.

## Discussion

### Principal Findings

In recent years, eHealth interventions have become an increasingly popular tool for supporting pharmacotherapy management due to the increasing pressure on health care systems and technological advancements. The digitalization of pharmacotherapy management was given a boost due to the SARS-CoV-2 pandemic. With this rapidly increasing amount of eHealth interventions, it is important to reflect on what interventions were successful and for what reasons, to guide future development.

This scoping review examined the use, effectiveness, and key factors for the success of eHealth interventions to improve pharmacotherapy management in 3 common chronic disease groups that frequently require careful pharmacotherapy management and are major contributors to polypharmacy (asthma or COPD, CVD, and diabetes). Our results show that eHealth interventions aim to improve pharmacotherapy management goals in the following 3 ways: medication adherence, treatment plan optimization, and—specifically for respiratory diseases—medication use. The effectiveness of eHealth interventions was mostly measured by clinician-reported or pharmacotherapy management outcomes. Patient-reported outcomes were less frequently used, with only one-half of the researched interventions doing so. Overall, 67% of included studies showed a positive effect. No trends were observed between the number of features or domains by Shaw et al [9] (Health in our Hands, Data Enabling Health, and Interacting for Health) and the effectiveness of the intervention, indicating that a more complex intervention does not necessarily lead to greater effects. However, feedback to the patient is suggested as a key factor for achieving a positive effect, particularly on clinician-reported outcomes.

We found that one-half of the eHealth interventions had a statistically significant positive effect on the pharmacotherapy management outcomes. A previous review demonstrated a similar effect (58% effective interventions) for eHealth interventions targeting medication adherence for adults with long-term medications [1]. Though this study had a broader scope of interventions, most eHealth interventions were focused on improving medication adherence. Medication therapies are effective to improve treatment outcomes for asthma or COPD, CVD, and diabetes, but long-term adherence is needed. However, nonadherence is common and has been associated with preventable morbidity and mortality and increased health care costs [74-76]. eHealth can be used as an opportunity for timely detection and addressing of nonadherence with limited time and resources.

Some of the included studies targeted this problem with advanced solutions that directly monitored the usage of medication with, for example, smart inhalers or ingestible sensors or by providing reminders to take medication [42,54,71,72].

However, the most prevalent strategy used by eHealth interventions to improve medication adherence was through monitoring of clinical measurements or disease-specific events, specifically focusing on parameters such as blood pressure for CVD, blood glucose for diabetes, and exacerbations for asthma or COPD. Although this does not directly target pharmacotherapy management, monitoring of these measurements can improve patients’ medication knowledge, motivation, and adherence. In addition, it provides health care providers with more insight into the patient’s adherence and the opportunity to motivate or adjust treatments where needed.

eHealth interventions are currently also used to target disease-specific pharmacotherapy management goals. Inhaled medications play a central role in the disease control of asthma and COPD. An incorrect inhaler technique with inhaler devices is common, which impacts the effectiveness of the medication and thereby leads to suboptimal disease control [77,78].

Of the total 16 studies conducted with patients with asthma or COPD, 38% (n=6) of them incorporated interventions aimed at improving medication use. Within this subset of studies, 5 demonstrated an overall positive impact [42,47,51,53]. The remaining studies aimed to investigate the viability of eHealth as a replacement for a non-eHealth standard of care. Although the effect of the eHealth intervention was equal to that of the standard of care, it was regarded as neutral in terms of overall impact [48].

Notably, our analysis revealed that most of the studies were designed with targeted methodologies for their eHealth interventions, specifically aimed at addressing pharmacotherapy management problems. In contrast, only a limited number of studies adopted a holistic approach by using the eHealth intervention to not only address pharmacotherapy management problems but also coach and gain insights on the patients’ overall healthy behaviors [23,29,38,44,65]. Given the relatively small number of studies following a holistic approach, we did not include a direct comparison between holistic and targeted interventions in our review. However, a noteworthy example of an intervention with a holistic approach was demonstrated by Persell et al [38]. In their study, a smartphone app was used to facilitate home monitoring and encourage behavioral changes associated with hypertension self-management. In addition to the ability to track blood pressure, the app also incorporated cognitive behavioral therapy techniques to provide support and coaching on various aspects of health related to hypertension, including diet, physical activity, sleep, and stress management. The app tailored the recommendations according to participants’ prior data and responses. It is interesting for further research to investigate whether holistically designed eHealth interventions lead to more sustainable solutions addressing health care challenges.

Shaw et al [9] stated “Of particular importance is the role of overlap among these domains for guiding the development of highly impactful innovations.” “Where all 3 domains overlap is the optimum point that integrates health data for enhancing interactions and communications to empower consumers to be active in their health and health care” [9] is suggested to be true by our results. In trying to uncover key factors for the success of an eHealth intervention, we indeed found that there is a trend toward more positive outcomes if the intervention had at least 2 or more overlapping domains versus only 1 domain. However, in the overall study outcomes, there was no clear difference in the added effect of 3 overlapping domains versus 2 overlapping domains, indicating that the added complexity of combining all 3 domains is not always needed to be successful.

For instance, in the study conducted by Zha et al [41], the eHealth intervention utilized all 3 domains. This intervention used a fully automated wrist-cuff blood pressure monitor in combination with a mobile app to measure blood pressure and pulse rate. The app tracked and analyzed the measurements, providing the patients with instant feedback, thereby facilitating self-monitoring and goal setting for ongoing improvement. However, the intervention did not lead to a significant improvement compared with the control group, mainly due to the already high standard of care in that population. The authors concluded that the impact of this eHealth intervention would be higher if it was applied with a population with a lower standard of care.

Conversely, our analysis revealed instances of eHealth interventions that solely focused on a single domain. For example, in the study by Egede et al [59], the eHealth device was only used for data collection by measuring blood glucose and blood pressure readings and transmitting these data to a secure website. A nurse case manager then would use these data to adjust medications if necessary. This intervention resulted in a significant improvement in HbA_1c_ levels.

Similarly, we evaluated whether interventions combining multiple features to improve pharmacotherapy management lead to better outcomes. Though there were more studies with 3 or more features showing a positive effect on pharmacotherapy management and patient-reported clinical outcomes, most interventions showing a positive effect on clinician-reported clinical outcomes had 2 features. This may be explained by the fact that pharmacotherapy management and patient-reported outcomes are often directly connected to the patient’s knowledge and motivation, for which more features may be needed to achieve a positive effect.

Though improvement of clinician-reported clinical outcomes is also shown to be strongly related to the patient’s motivation, these outcomes can generally be measured directly from the patient (eg, blood pressure). Therefore, large effects may be achieved with fewer features. Since differences were marginal for all 3 outcomes and the fact that clinician-reported clinical outcomes were mostly positive for interventions with just 2 features, we conclude there is no clear connection between the number of features and the effectiveness of the eHealth interventions and was therefore not considered a key contributor to successful interventions.

Last, we examined if feedback to the patient could be considered a key factor for successful interventions. In the overall effect on outcomes, no clear trends were observed between interventions that did provide feedback to patients and those that did not. However, when specifically looking at the clinician-reported clinical outcomes, 66% of the interventions that provided feedback had a positive effect, whereas only 13% of interventions had a positive effect when patients did not receive feedback. Data provided by the eHealth intervention create the opportunity for caregivers to provide the patient with personalized feedback and thus educate the patient on how to improve their pharmacotherapy management or to support them on good behavior and thus stimulate the patient to maintain this. Also, feedback can help patients to better understand their self-measurements and conclude if it is within the desired range and allows them to reflect on their personal disease management. Results of previous reviews support that feedback can be seen as a key factor for successful eHealth interventions [1,79].

Further research is needed to determine if feedback is indeed a key component for successful eHealth interventions. The divergent findings underline the importance of considering the specific context and health care landscape while developing eHealth interventions. An effective intervention is one that is tailored to the needs and requirements of the target population. As the field of eHealth continues to evolve rapidly, it remains essential to notice the nuances between intervention designs, considering the population’s characteristics, existing health care infrastructure, and other unique challenges. By gaining a deeper understanding of these considerations, we can advance toward the development of truly impactful and patient-centered eHealth interventions.

### Limitations

A limitation of this study is that it is only focused on 3 disease categories. There are a lot more diseases for which treatment relies on careful pharmacotherapy management and eHealth interventions are applied and researched. However, to ensure the feasibility of the systematic review, the population was limited to the 3 main indications with chronic medication use. Though we found that the design of the eHealth intervention strongly depends on the type of disease, optimal pharmacotherapy management can often be achieved with the same 3 principles: improving medication adherence, medication use, and optimization of the treatment plan. These are principles from which all diseases that require long-term medication use could benefit; thus, we think the results also have implications for other chronic disease categories.

Moreover, we found that there is a lot of variety in the outcome measures used. The heterogeneity in outcome measures was also found in other reviews on the effect of eHealth on medication adherence [1,80]. We found similar heterogeneity in the clinician- and patient-reported outcome measures. For these reasons, it is difficult to compare the effectiveness of the eHealth interventions and should therefore be treated with caution. Future research could benefit from using standardized clinical and medication adherence outcome measurements within disease categories.

### Conclusions

A wide variety of interventions has been developed, combining various domains and features to target pharmacotherapy management for patients with asthma or COPD, CVD, and diabetes. Most eHealth interventions have an overall positive effect. For the development of eHealth interventions with outcomes that rely on clinician-reported measurements, our results suggest a feedback mechanism as a key factor for success. Future research should be done to examine which mechanisms of feedback are most effective. The results also suggest that eHealth interventions become more impactful when overlapping 2 or 3 eHealth domains. These insights can aid future eHealth intervention development.

## Appendix

Multimedia Appendix 1 Protocol.

Multimedia Appendix 2 Final Embase search strategy.

Multimedia Appendix 3 Included articles.

## Abbreviations

COPD: chronic obstructive pulmonary disease
CVD: cardiovascular disease
PICOS: Population, Intervention, Comparator, Outcomes, Study Design
PRISMA-ScR: Preferred Reporting Items for Systematic Reviews and Meta-Analyses extension for scoping reviews
RCT: randomized controlled trial
