# Supplementary material for: The Use of eHealth for Pharmacotherapy Management With Patients With Respiratory Disease, Cardiovascular Disease, or Diabetes: Scoping Review
Source: J Med Internet Res. 2023 Sep 26;25:e42474. doi: 10.2196/42474 (PMC10565624; doi:10.2196/42474)
Supplement: Multimedia Appendix 1 [file jmir_v25i1e42474_app1.docx]

**Scoping Review Protocol for the use of eHealth for pharmacotherapy management in patients with respiratory disease, cardiovascular disease, or diabetes**

Protocol

18 February 2020

Updated 1 October 2021

## Abbreviations

|  |  |
| --- | --- |
| COPD | Chronic Obstructive Pulmonary Disease |
| eHealth | Electronic Health |
| MeSH | Medical Subject Headings |
| PICOS | Population, Intervention, Comparator, Outcomes, Study design |
| PRISMA | Reporting Items for Systematic Reviews and Meta-Analyses |
| RCT | Randomised Clinical Trial |
|  |  |
|  |  |
|  |  |
|  |  |
|  |  |
|  |  |
|  |  |
|  |  |
|  |  |
|  |  |
|  |  |
|  |  |
|  |  |
|  |  |
|  |  |
|  |  |
|  |  |
|  |  |
|  |  |
|  |  |
|  |  |
|  |  |
|  |  |
|  |  |
|  |  |
|  |  |
|  |  |
|  |  |
|  |  |

## Introduction and rationale

A commonly used definition of eHealth is: “health services and information, delivered through the internet and related information and communication technologies, to improve or to enable health and healthcare” [1]. eHealth is viewed as an important tool to solve the problem of the increasing demand that healthcare systems are facing due to an aging population [2]. It is believed that with the use of eHealth, healthcare can remain accessible and of good quality, and also introduce more patient empowerment.

Community pharmacies play an important role in the safe and effective use of drugs of patients in ambulatory settings. eHealth can help the pharmacist to improve the efficiency of healthcare by providing the patient with easily accessible information. Also, pharmacists expect that eHealth may lower the threshold to contact the pharmacy [3], which may also contribute to efficient healthcare.

Although there is a growing interest in eHealth to support patients and healthcare providers in disease management, the question remains what type of interventions are most effective. eHealth is a broad concept, and many different types of interventions may be developed. However, development and implementation is time consuming and costly. Therefore, it is relevant to explore the possibilities and limitations of eHealth interventions in the community pharmacy published to this point and look for lessons learned.

With this scoping literature review we aim to explore on an international level how eHealth is currently used for pharmacotherapy management in patients with respiratory disease, cardiovascular disease, or diabetes.

## Objective

A scoping review will be performed to identify and describe all relevant of eHealth interventions in community pharmacy described in literature.

The specific objectives of the scoping review are as follows:

- To systematically review and describe the body of literature that exists on eHealth interventions in the public pharmacy.
- To critically appraise all relevant studies and answer the research questions based on the PICOS.
- To prepare a draft manuscript for publication in an international peer-reviewed journal.

With the scoping review we aim to answer the following research questions:

- How is eHealth used for pharmacotherapy management in patients with asthma/COPD, CVD, and diabetes? and
- How effective are eHealth interventions on pharmacotherapy management and clinical outcomes? Subsequently, the type of intervention was linked to the effectiveness to answer the third question
- What key aspects make eHealth interventions for pharmacotherapy management successful?

## Definition of eHealth

Because there is not a standard definition of eHealth, the choice of definition could affect the results of the scoping review. Therefore, we predefine the definition of eHealth as described by Nictiz and Nivel in the e-health monitor of 2019 [4] as: “E-health is defined as the use of digital information as well as digital communication to support and/or improve health and healthcare”. However, this definition is broad and not very specific. Therefore, we combine this definition with the conceptual model of Shaw et al. [5] to make the definition more usable for the scoping review. This conceptual model is based on semi-structured interviews with key informants (n=25) and contains three thematically analysed emergent domains (see Figure 1):

1. Health in our hands (using eHealth technologies to monitor, track, and inform healthcare),
2. Interacting for health (using digital technologies to enable health communication among practitioners and between health professionals and clients or patients), and
3. Data enabling health (collecting, managing, and using health data).

The PICOS and in- and exclusion criteria of the scoping review are based on a combination of the definition from the e-health monitor 2019 and the defined domains by Shaw et al. [5], with the intervention fitting at least in 1 of the 3 domains.


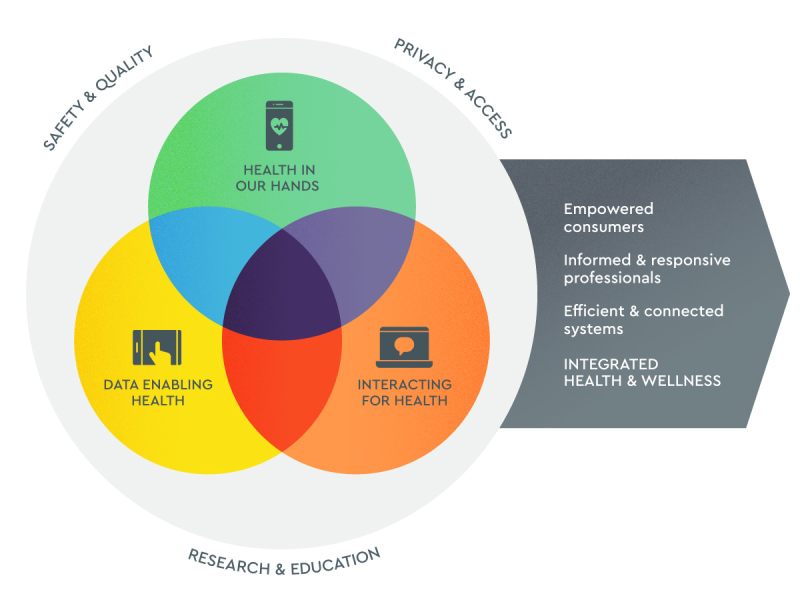


Figure 1. The conceptual model of Shaw et al. [5]. Figure reproduced with permission from Shaw et al. (2017).

## Methods

### Electronic databases

The searches for the scoping review will be designed with a combination of sensitivity and specificity. The following electronic databases will be searched:

- Embase (embase.com)
- MEDLINE (PubMed.com)
- The Cochrane Library, including:
  - The Cochrane Database of Systematic Reviews (CDSR)
  - The Cochrane Central Register of Controlled Trials (CENTRAL)

The pilot search strategy for the scoping review is presented in Table 1. The Embase search strategy will be adapted to search the other electronic databases.

Table 1. Search query used for the scoping review.

| **Electronic database** | **Search terms** |
| --- | --- |
| MEDLINE (Pubmed) | 'community pharmacist'/exp OR 'community pharmacist' OR 'community pharmacist':ab,ti,kw OR 'pharmacy (shop)'/exp OR 'pharmacy (shop)' OR 'pharmacy (shop)':ab,ti,kw OR 'pharmacist':ab,ti,kw OR 'pharmacists':ti,ab,kw OR 'pharmacies':ti,ab,kw OR 'pharmacy':ti,ab,kw OR 'medication compliance'/exp OR 'medication compliance' OR 'medication compliance':ti,ab,kw OR 'medication therapy management'/exp OR 'medication therapy management' OR 'medication therapy management':ti,ab,kw  AND  'telehealth'/exp OR 'telehealth':ab,ti,kw OR 'tele-health':ab,ti,kw OR 'telemedicine'/exp OR 'telemedicine':ab,ti,kw OR 'tele-medicine':ab,ti,kw OR 'ehealth':ab,ti,kw OR 'e-health':ti,ab,kw OR 'mobile health application':ab,ti,kw OR 'mhealth':ab,ti,kw OR 'm-health':ab,ti,kw OR 'telemonitoring'/exp OR 'telemonitoring':ab,ti,kw OR 'tele-monitoring':ab,ti,kw OR 'digital health':ti,ab,kw AND 'clinical article'/de OR 'clinical study'/de OR 'clinical trial'/de OR 'cohort analysis'/de OR 'comparative effectiveness'/de OR 'comparative study'/de OR 'controlled clinical trial'/de OR 'controlled study'/de OR 'cross sectional study'/de OR 'evidence based#1 and #2 medicine' OR 'evidence based practice'/de OR 'feasibility study'/de OR 'intention to treat analysis'/de OR 'intervention study'/de OR 'longitudinal study'/de OR 'major clinical study'/de OR 'meta analysis'/de OR 'multicenter study'/de OR 'observational study'/de OR 'pilot study'/de OR 'prospective study'/de OR 'qualitative research'/de OR 'quantitative study'/de OR 'randomized controlled trial'/de OR 'randomized controlled trial topic'/de OR 'retrospective study'/de OR 'systematic review'/de OR 'questionnaire'/de OR 'teleconsultation'/de OR 'telephone interview'/de OR 'transitional care'/de  NOT  'clinical audit'/de OR 'clinical protocol'/de OR 'drug surveillance program'/de OR 'interview'/de OR 'medical record review'/de OR 'model'/de OR 'semi structured interview'/de OR 'simulation'/de OR 'total quality management'/de  AND  [english]/lim |
| Embase | 'community pharmacist'/exp OR 'community pharmacist':ab,ti,kw OR 'pharmacy (shop)'/exp OR 'pharmacy (shop)' OR 'pharmacy (shop)':ab,ti,kw OR 'pharmacist':ab,ti,kw OR 'pharmacists':ti,ab,kw OR 'pharmacies':ti,ab,kw OR 'pharmacy':ti,ab,kw  AND  'telehealth'/exp OR 'telehealth':ab,ti,kw OR 'tele-health':ab,ti,kw OR 'telemedicine'/exp OR 'telemedicine':ab,ti,kw OR 'tele-medicine':ab,ti,kw OR 'ehealth':ab,ti,kw OR 'e-health':ti,ab,kw OR 'mobile health application':ab,ti,kw OR 'mhealth':ab,ti,kw OR 'm-health':ab,ti,kw OR 'telemonitoring'/exp OR 'telemonitoring':ab,ti,kw OR 'tele-monitoring':ab,ti,kw  AND  'clinical article'/de OR 'clinical study'/de OR 'clinical trial'/de OR 'cohort analysis'/de OR 'comparative effectiveness'/de OR 'comparative study'/de OR 'controlled clinical trial'/de OR 'controlled study'/de OR 'cross sectional study'/de OR 'evidence based#1 and #2 medicine' OR 'evidence based practice'/de OR 'feasibility study'/de OR 'intention to treat analysis'/de OR 'intervention study'/de OR 'longitudinal study'/de OR 'major clinical study'/de OR 'meta analysis'/de OR 'multicenter study'/de OR 'observational study'/de OR 'pilot study'/de OR 'prospective study'/de OR 'qualitative research'/de OR 'quantitative study'/de OR 'randomized controlled trial'/de OR 'randomized controlled trial topic'/de OR 'retrospective study'/de OR 'systematic review'/de OR 'questionnaire'/de OR 'teleconsultation'/de OR 'telephone interview'/de OR 'transitional care'/de  NOT  'clinical audit'/de OR 'clinical protocol'/de OR 'drug surveillance program'/de OR 'interview'/de OR 'medical record review'/de OR 'model'/de OR 'semi structured interview'/de OR 'simulation'/de OR 'total quality management'/de  AND  [english]/lim |
| Cochrane Library | MeSH descriptor: [Pharmacists] explode all trees OR MeSH descriptor: [Pharmacies] explode all trees OR 'pharmacist':ab,ti,kw OR 'pharmacists':ti,ab,kw OR 'pharmacies':ti,ab,kw OR 'pharmacy':ti,ab,kw OR 'community pharmacist':ti,ab,kw  AND  MeSH descriptor: [Telemedicine] explode all trees OR MeSH descriptor: [Mobile Applications] explode all trees OR 'telehealthcare':ti,ab,kw OR 'telehealth':ti,ab,kw OR 'tele-health':ti,ab,kw OR 'telemedicine':ti,ab,kw OR 'tele-medicine':ti,ab,kw OR 'ehealth':ti,ab,kw OR 'e-health':ti,ab,kw OR 'mhealth':ti,ab,kw OR 'm-health':ti,ab,kw OR 'telemonitoring':ti,ab,kw OR 'tele-monitoring':ti,ab,kw OR 'mobile applications':ti,ab,kw  *in Cochrane Reviews and Trials |

Reference lists of any identified trials, reviews or meta analyses of eHealth interventions will be searched for further studies of interest, because such reference lists typically are good sources of additional material that can supplement the articles identified in the medical literature databases.

### Other sources

Next to the electronic databases Cochrane reviews on e-health will be checked for relevant articles eligible for inclusion in our scoping review. We will also conduct bibliographic searches of identified key systematic review and meta-analysis (including network-meta analysis) articles to ensure that initial searches captured all the relevant clinical studies.

### Time horizon and limitations

The electronic database searches will be performed without a date, geographical or study design limitation. The search in the electronic medical databases will be limited to the language English.

### Search terms

Search terms will include combinations of free text and Medical Subject Headings (MeSH) or Emtree subject headings. The following concepts will be included in the search strategy:

- Search terms relating to the population of interest
- Exclusionary terms: unwanted publication types (e.g., terms for comments, editorials, letters, and case reports) and studies in animals

The MEDLINE search strategy will be adapted to search other electronic databases. Search terms for the searches to be performed in the online resources will be drawn from the listings in the appendix, as appropriate for the search features of individual sites. A log of these searches will be maintained in Excel.

### Study selection criteria

Potentially relevant publications will be reviewed and assessed to collate a final set of studies that will be used to answer to the research questions. To determine the final set of studies eligible for review, explicit inclusion and exclusion criteria will be applied to the literature search results. The inclusion and exclusion criteria are specified in Table 2.

**Table 2. PICOS criteria for the inclusion and exclusion criteria of studies.**

| **Category** | **Inclusion criteria** | **Exclusion criteria** |
| --- | --- | --- |
| Population (P) | - Asthma/COPD patients - Cardiovascular patients - Diabetes Mellitus patients | - |
| Intervention (I) | eHealth interventions covering at least one of the three domains by Shaw et al. and directly or indirectly supports pharmacotherapy management including medication adherence. | - |
| Comparators (C) | Usual care | No comparator  Versus Baseline |
| Outcomes (O) ^b^ | - Pharmacotherapy management or clinical outcomes | - |
| Study design (S) | - RCTs – both parallel-group and crossover (double-blind, single-blind, open-label) - Retrospective and prospective cohort studies | - In vitro studies - Preclinical studies - Comments, letters and editorials - Case reports, case series - Guidelines - Single arm trials - Review - Meta analysis |
| Language | English | Non-English |
| Time limit | No restriction | - |
| Country | Western countries:  Europe  United Kingdom  United States  Canada  Australia  New Zealand | - |
| Note: If it is unclear whether a study meets any criterion during the level 1 screening process, the study will be progressed to full-text screening to confirm its inclusion in the review.  ^a^ Non comparative studies will be excluded  ^b^ Outcome criteria will be used at level 2 screening, used to answer research question 2 | | |

### Study selection methodology

All retrieved studies will be assessed against the study selection criteria for the search. The study selection process will be performed in the following two phases:

- Primary (Level 1) screen: titles and abstracts of studies identified from the electronic databases will be double screened by two independent researchers to determine eligibility according to the inclusion and exclusion criteria described in Table 2. If there is disagreement about study relevance, consensus can be reached through a discussion.
- Secondary (Level 2) screen: full texts of studies selected at level 1 will be obtained and double screened by two independent researchers to determine eligibility according to the inclusion and exclusion criteria described in Table 2. If there is disagreement about study relevance, consensus will be reached through a third researcher.

This inclusion and exclusion process will be documented, including completion of a Preferred Reporting Items for Systematic Reviews and Meta-Analyses (PRISMA) flow diagram (Figure 2) within the draft manuscript. This documentation will detail the number of articles included or excluded at each screening level. A list of studies excluded at level 1 and level 2, and the reason for each exclusion, will be developed in an Excel worksheet.

Identification

Screening

Eligibility

Included

Number of records identified through database search

(Embase = n, MEDLINE = n, Cochrane Library = n)

Number of additional records identified through other sources

Number of records after duplicates removed

Level 1

Number of records screened by abstract

Level 1

Number of records excluded

Level 2

Number of full-text articles assessed for eligibility

Level 2

Number of full-text articles excluded, with reasons

Number of studies included in qualitative synthesis

**Figure 2. PRISMA flow diagram [6].**

## Data extraction

Data will be extracted from full-text publications where available (i.e., we will not use abstracts or posters unless an abstract or poster is the terminal source document). References to other publications within a study will be traced to original sources where appropriate. A standardized evidence data extraction table shell will be developed in Microsoft Excel and will populate this shell with the information extracted from the included studies. Before starting data extraction, the proposed extraction table shell will be shared with the research team to receive feedback and approval. Once received, data extraction will be started. Data extraction will be carried out by one researcher. However, any uncertainty about the data will be resolved by discussion with another researcher. Quality-control procedures will include verification of all extracted data with original sources.

### Study and patient characteristics

Data regarding study characteristics, patient demographics, interventions, efficacy/effectiveness outcomes, found in the included study will be extracted by a single reviewer from full-text versions of studies where available. The extracted data will be tabulated. Tables 3 present example table shells. The following is a non-exhaustive list of some of the important study and patient characteristics to be extracted included in the data extraction template:

- Study design and characteristics
  - Type of study
  - Description of the study
- Intervention
  - Description of intervention
  - Domains of conceptual model from Shaw et al. [5]
- Comparator
  - Description of comparator
- Patient characteristics
  - Number of patients
  - Population description (ethnicity, age, disease)
  - Inclusion/exclusion criteria
- Efficacy outcomes
  - Results summary
  - Overall effect (negative, neutral, positive)
  - Effect on medication management
  - Effect on clinical outcomes

**Table 3. Summary table shell example for clinical studies (study details)**

| **Citation** | **Study design** | **Study description** | **Intervention description** | **Domains of Shaw et al.** | **Comparator** | **Number of patients** | **Population description** | **Inclusion criteria** | **Exclusion criteria** | **Study results** | **Overall effect ^a^** | **Effect medication management** | **Effect clinical outcomes** |
| --- | --- | --- | --- | --- | --- | --- | --- | --- | --- | --- | --- | --- | --- |
|  |  |  |  |  |  |  |  |  |  |  |  |  |  |

^a^ Negative, neutral, positive

## Risk of bias (quality) assessment of extracted studies

Since this is a scoping review, we will not conduct risk of bias assessment, which is consistent with the framework proposed by the Joanna Briggs Institute and PRISMA methodological guidelines for Scoping reviews [7,8].

## Reporting

The findings of the scoping review will be described in a draft manuscript. The manuscript will include details of the findings of the literature search (PRISMA flow diagram), the study selection process, and tables/figures for extracted data. Additionally, the report will summarize the general trends of the evidence base in a descriptive manner.

## References

1. Eysenbach G. What is e-health? Journal of Medical Internet Research; 2001;3(2):1–5. doi: 10.2196/jmir.3.2.e20.

2. Ross J, Stevenson F, Lau R, Murray E. Factors that influence the implementation of e-health: a systematic review of systematic reviews (an update). Implement Sci; 2016 Dec;11(1):146. doi: 10.1186/s13012-016-0510-7.

3. van Lettow B, Schreuder C, Custers B, Ottenheijm S, Krijgsman J. eHealth, de apotheker is er klaar voor. Den Haag; 2016. Cited 2020 Jan 28. <https://www.nictiz.nl/wp-content/uploads/2016/06/Rapport_eHealth-de-apotheker-is-er-klaar-voor.pdf>

4. Nictiz and Nivel. eHealth-monitor 2019 Rapport. 2019 Nov. Cited 2020 Feb 5. <http://www.nictiz.nl/rapporten/ehealth-monitor-2019-rapport/>

5. Shaw T, McGregor D, Brunner M, Keep M, Janssen A, Barnet S. What is eHealth (6)? Development of a Conceptual Model for eHealth: Qualitative Study with Key Informants. J Med Internet Res. 2017 Oct 24;19(10):e324. doi: 10.2196/jmir.8106.

6. PRISMA. PRISMA 2009 Flow Diagram. Vol. 6, The PRISMA statement. 2009. Cited 2019 Sep 4. p. 1000097. <http://www.prisma-statement.org/PRISMAStatement/FlowDiagram.aspx>

7. Peters MDJ, Godfrey C, McInerney P, Munn Z, Tricco AC, Khalil H. Chapter 11: Scoping Reviews (2020 version). Inst Rev Manual; JBI. 2020; <https://reviewersmanual.joannabriggs.org/>

8. Tricco, AC, Lillie, E, Zarin, W, O’Brien, KK, Colquhoun, H, Levac, D, Moher, D, Peters, MD, Horsley, T, Weeks, L, Hempel S et al. PRISMA extension for scoping reviews (PRISMA-ScR): checklist and explanation. Ann Intern Med; 2018;169(7):467–73. doi: 10.7326/M18-0850.
