# Supplementary material for: The Use of eHealth for Pharmacotherapy Management With Patients With Respiratory Disease, Cardiovascular Disease, or Diabetes: Scoping Review
Source: J Med Internet Res. 2023 Sep 26;25:e42474. doi: 10.2196/42474 (PMC10565624; doi:10.2196/42474)
Supplement: Multimedia Appendix 2 [file jmir_v25i1e42474_app2.docx]

## **Appendix 2 – Final Embase search strategy**

**Table 1. Search strategy Embase (March 23, 2020; update October 1, 2021).**

| #1 | 'community pharmacist'/exp OR 'community pharmacist' OR 'community pharmacist':ab,ti,kw OR 'pharmacy (shop)'/exp OR 'pharmacy (shop)' OR 'pharmacy (shop)':ab,ti,kw OR 'pharmacist':ab,ti,kw OR 'pharmacists':ti,ab,kw OR 'pharmacies':ti,ab,kw OR 'pharmacy':ti,ab,kw OR 'medication compliance'/exp OR 'medication compliance' OR 'medication compliance':ti,ab,kw OR 'medication therapy management'/exp OR 'medication therapy management' OR 'medication therapy management':ti,ab,kw |
| --- | --- |
| #2 | 'telehealth'/exp OR 'telehealth':ab,ti,kw OR 'tele-health':ab,ti,kw OR 'telemedicine'/exp OR 'telemedicine':ab,ti,kw OR 'tele-medicine':ab,ti,kw OR 'ehealth':ab,ti,kw OR 'e-health':ti,ab,kw OR 'mobile health application':ab,ti,kw OR 'mhealth':ab,ti,kw OR 'm-health':ab,ti,kw OR 'telemonitoring'/exp OR 'telemonitoring':ab,ti,kw OR 'tele-monitoring':ab,ti,kw OR 'digital health':ti,ab,kw |
| #3 | #1 AND #2 |
| #4 | 'clinical article'/de OR 'clinical study'/de OR 'clinical trial'/de OR 'cohort analysis'/de OR 'comparative effectiveness'/de OR 'comparative study'/de OR 'controlled clinical trial'/de OR 'controlled study'/de OR 'cross sectional study'/de OR 'evidence based#1 and #2 medicine' OR 'evidence based practice'/de OR 'feasibility study'/de OR 'intention to treat analysis'/de OR 'intervention study'/de OR 'longitudinal study'/de OR 'major clinical study'/de OR 'meta analysis'/de OR 'multicenter study'/de OR 'observational study'/de OR 'pilot study'/de OR 'prospective study'/de OR 'qualitative research'/de OR 'quantitative study'/de OR 'randomized controlled trial'/de OR 'randomized controlled trial topic'/de OR 'retrospective study'/de OR 'systematic review'/de OR 'questionnaire'/de OR 'teleconsultation'/de OR 'telephone interview'/de OR 'transitional care'/de |
| #5 | #3 AND #4 |
| #6 | 'clinical audit'/de OR 'clinical protocol'/de OR 'drug surveillance program'/de OR 'interview'/de OR 'medical record review'/de OR 'model'/de OR 'semi structured interview'/de OR 'simulation'/de OR 'total quality management'/de |
| #7 | #5 NOT #6 |
| #8 | [english]/lim |
| #9 | #7 AND #8 |
